# Management of Syncope in Geriatric Patients: Are 2018 European Society of Cardiology Guidelines Still Relevant?

**DOI:** 10.3390/jcm15166303

**Published:** 2026-08-14

**Authors:** Barbara Antonazzo, Anna Sirignano, Martina Salvini, Francesca Flavia Rossi, Stefano Ronzoni, Agnese Dorizzi, Kenneth Ellenbogen, Giuseppe Biondi-Zoccai, Simone Calcagno

**Affiliations:** 1 Division of Geriatrics, Israelite Hospital, 00148 Rome, Italy; barb.antonazzo@gmail.com (B.A.); fr.rossi@ospedaleisraelitico.it (F.F.R.); s.ronzoni@ospedaleisraelitico.it (S.R.); a.dorizzi@ospedaleisraelitico.it (A.D.); 2 Division of Cardiology, Santa Maria Goretti Hospital, 04100 Latina, Italy; an.sirignano@gmail.com; 3 Faculty of Medicine and Surgery, Sapienza University of Rome, 04100 Latina, Italy; salvini.1938973@studenti.uniroma1.it; 4 Division of Cardiology, VCU School of Medicine, Richmond, VA 23298, USA; kenneth.ellenbogen@vcuhealth.org; 5 Department of Medical-Surgical Sciences and Biotechnologies, Sapienza University of Rome, 04100 Latina, Italy; gbiondizoccai@gmail.com; 6 IRCCS Maria Cecilia Hospital—GVM Care & Research, 48033 Cotignola, Italy; 7 Cardiology Unit, Department of Emergency and Admission, San Paolo Hospital, Largo Donatori del Sangue 1, 00053 Civitavecchia, Italy

**Keywords:** elderly, guidelines, syncope, healthy aging, digital medicine, telemedicine

## Abstract

Syncope in older adults is common, heterogeneous, and frequently misclassified as an unexplained fall, leading to injury, fear of recurrence, and costly, low-yield testing and hospitalization. The 2018 European Society of Cardiology (ESC) Guidelines emphasize structured initial evaluation (history with witnesses, orthostatic blood pressure, and 12-lead ECG), risk-feature-guided disposition, and targeted second-line investigations rather than routine neuroimaging or carotid duplex ultrasound. We performed a structured narrative review anchored to the ESC 2018 Guidelines, searching MEDLINE/PubMed up to February 2026 for randomized trials, observational cohorts, and systematic reviews with substantial representation of adults aged ≥65 years and extracting geriatric-relevant outcomes including injury, falls, admission, arrhythmic diagnoses, and short-term serious events. Evidence since 2018 supports the enduring value of the ESC 2018 Guidelines’ framework but highlights the need for geriatric adaptation: repeated orthostatic phenotyping (including post-prandial and delayed hypotension), systematic deprescribing, and explicit integration of frailty, cognition, and trauma risk into disposition decisions. Post-2018 data strengthen mechanism-based strategies, including earlier implantable loop recorders with remote monitoring and selective pacing for tilt-induced asystolic reflex syncope, while cardioneuroablation remains investigational for most elders. We propose a pragmatic pathway prioritizing diagnostic yield, minimizing low-value testing, and aligning interventions with patient goals and functional outcomes to reduce recurrences and support safer transitions.

## 1. Introduction

Syncope in older adults is common, heterogeneous, and frequently misclassified as an unexplained fall, with consequences that range from injury and risk of recurrence to unnecessary testing and hospitalization [1]. Age-related autonomic impairment, polypharmacy, and multimorbidity blur classic prodromes, while cognitive dysfunction limits history quality, so diagnostic confidence often depends on structured bedside assessment and witness information [2].

The 2018 European Society of Cardiology (ESC) Guidelines prioritize initial evaluation with orthostatic blood pressure measurement and 12-lead ECG, then risk-feature-guided disposition and targeted testing rather than routine neuroimaging or carotid ultrasound [3]. In geriatric wards, however, thresholds for “high-risk” may be crossed by prevalent baseline abnormalities, and care teams must reconcile guideline pathways with frailty, limited physiologic reserve, and competing goals of care [4].

Since 2018, pragmatic data on implantable loop recorders (ILRs), remote monitoring, and pacing for tilt-induced asystolic reflex syncope have expanded, alongside renewed interest in cardioneuroablation for selected patients [5]. Concurrently, multiple emergency-department risk scores have been validated with variable performance, prompting debate about whether such tools add value beyond expert clinical judgement in older, comorbid populations [6].

This review focuses on strengths and weaknesses of the ESC 2018 Guidelines in geriatrics, aiming also at identifying areas needing adaptation, and synthesizing post-2018 evidence relevant to orthostatic phenotyping, monitoring strategy, and mechanism-based therapy. We also aim at providing a pragmatic framework suitable to reduce low-yield testing, improving diagnostic yield, and aligning interventions with patient priorities, with the goal of reaching more appropriate decisions and thus fewer recurrences.

## 2. Methods and Scope of Evidence Synthesis

We conducted a structured narrative review anchored to the ESC 2018 Guidelines on syncope, aiming to evaluate their applicability in geriatric care across emergency, inpatient, and outpatient settings during the post-2018 era [7]. Searches were performed in MEDLINE/PubMed for the period January 2018 to February 2026 with Boolean logic for relevant work matching the following keywords: elderly, older adults, geriatric, aged, orthostatic hypotension, falls, vasovagal, frail or fragility elderly, pseudosyncope and loss of consciousness. In addition to electronic database searches via PubMed, a comprehensive literature retrieval strategy was implemented to minimize selection bias. We performed handsearching across the official websites of key peer-reviewed journals in the fields of geriatrics and cardiology. Furthermore, citation tracking was utilized through both backward and forward snowballing techniques; the reference lists of all relevant articles were screened retrospectively to identify historical or foundational studies, while forward citation tracking was conducted using Google Scholar to capture recent publications that cited the selected literature. We focused on randomized trials, observational cohorts, and systematic reviews addressing adults aged ≥ 65 years or cohorts with older representation, evaluating diagnostic strategies, tools, or therapies for reflex, orthostatic, or cardiac syncope. Studies focused exclusively on pediatric populations, isolated seizure disorders, or non-transient loss of consciousness were excluded, while mixed transient loss of consciousness (TLOC) studies were retained when syncope-specific analyses, predictors, or outcomes were extractable. Data extraction prioritized patient age, frailty or cognitive impairment descriptors, setting, index evaluation components, and outcomes relevant to geriatrics, including injury, falls, hospitalization, arrhythmic diagnoses, and 30-day serious events where reported.

We mapped each evidence block to the corresponding ESC 2018 Guidelines, highlighting concordance, uncertainty, and situations common in older adults, such as polypharmacy, orthostatic hypotension, and frequent unwitnessed falls. As heterogeneity precluded pooled estimates for several questions, we described findings qualitatively, giving higher relevance to external validations, multicenter cohorts, and randomized evidence, and we noted implementation barriers within geriatric care pathways.

## 3. Syncope in Older Adults: Epidemiology, Mechanisms, and Phenotypes

Syncope accounts for a substantial share of emergency visits and admissions among older adults, yet its incidence is underestimated because events are unwitnessed, labelled as falls, or obscured by cognitive impairment and polypharmacy in care datasets [8]. Compared with younger patients, older individuals have higher rates of injury, hospitalization, and short-term adverse outcomes, largely driven by comorbid cardiovascular disease and frailty; nevertheless, benign reflex or orthostatic mechanisms still dominate many presentations in contemporary geriatric acute-care pathways [1,9].

Aging alters autonomic reserve, vascular compliance, and baroreflex sensitivity, predisposing to hypotensive susceptibility, post-prandial drops, and medication-related blood pressure lability; dehydration, anemia, and infection further lower the threshold for transient cerebral hypoperfusion during activities and positional changes [10]. Cardiac causes become more frequent with age, including bradyarrhythmias, conduction disease, and structural lesions, but attribution is challenging because incidental ECG abnormalities are common [1,2]. Accordingly the ESC 2018 Guidelines appropriately emphasize correlating symptoms with hemodynamics or rhythm documentation before definitive interventions.

In a more recent European Clinical Consensus Statement on mental health and cardiovascular disease, the authors introduced a focus on management of psychotropic drugs with QT interval prolongation properties that onset with syncope [11]. Considering the large use of psychotropic drugs in this setting of older patients and its correlation with arrhythmias, it is important to exclude ECG alteration. In such document, the authors propose a decision algorithm for the management of psychotropic drugs with ECG alterations could be the cause of a syncope. The diagnostic workup suggests a 12-lead ECG providing QTc at baseline and after 1, 6, and 12 weeks after initiating drug and ECG more often in case of QT prolongation and electrolytes abnormalities. In the text it is underlined the necessity to evaluate the link between drug use and syncope and, if clinically suspected, switching to other drugs with less propensity to cause changes in heart rhythm may be appropriate.

Phenotypically, geriatric syncope often presents as unexplained falls, near-syncope, or prolonged post-event confusion and may be multifactorial, combining orthostatic hypotension with reflex triggers or arrhythmias; systematic medication review is integral to classification and to preventing recurrence in frail patients [1,12]. Recognizing distinct phenotypes, such as neurogenic orthostatic hypotension, carotid sinus syndrome, vasovagal syncope with cardioinhibition, and arrhythmic syncope, guides targeted testing such as active standing, carotid sinus massage, tilt testing, and early loop recording when episodes recur or when high-risk features persist clinically [13].

## 4. Methodological Continuity and the Contemporary Clinical Utility of the 2018 ESC Guidelines in Geriatric Syncope Management

Nearly a decade after their publication, the 2018 European Society of Cardiology (ESC) Guidelines for the diagnosis and management of syncope continue to provide an invaluable, highly durable framework for clinical practice in same aspects. In geriatrics medicine the diagnostic approach is heavily challenged by multi-system aging, cognitive blurring, and atypical clinical presentations. Therefore, a diagnostic success in a structured, multi-component initial evaluation remains crucial. In daily geriatric care, the cornerstone of this assessment remains a meticulous clinical history that incorporates eyewitness accounts—essential for overcoming age-related retrograde amnesia—alongside a focused physical examination, standard orthostatic blood pressure measurements, and an immediate 12-lead electrocardiogram (ECG). This fundamental approach successfully identifies the underlying syncope mechanism early in a substantial proportion of frail older individuals.

Furthermore, the guidelines’ emphasis on clinical pattern recognition remains highly effective for older adults. Explicit diagnostic criteria utilize specific situational triggers and characteristic autonomic prodromes to identify reflex syncope, although these warnings are frequently shortened or absent in older cohorts. Clear postural timing and reproducible orthostatic drops confirm orthostatic hypotension (OH), which represents a major cause of transient loss of consciousness (TLOC) in the elderly. Conversely, episodes occurring during exertion or while supine or accompanied by specific ECG red flags—such as bifascicular block, persistent sinus bradycardia, or non-sustained ventricular arrhythmias—highly suggest a life-threatening cardiac etiology that requires immediate protective intervention.

The guidelines also underline the importance of early risk classification of syncope. Also this aspect remain valid and applicable. The protocol-driven triage of older patients in emergency settings relies on identifying specific high- and low-risk clinical features to dictate appropriate pathways. Low-risk features safely support direct discharge from the emergency department (ED). High-risk traits—such as unexplained hypotension, severe structural heart disease, or trauma secondary to the event—dictate intensive evaluation within a specialized syncope unit or require immediate hospital admission. Crucially, the guidelines underscore that commercial risk-stratification scores do not outperform sound clinical judgment. This caution is highly relevant in geriatrics, where complex comorbidities and frailty can distort rigid scoring systems. The guidelines also promote diagnostic parsimony by establishing that routine, unselected testing is low-yield. Advanced neuroimaging, head computed tomography, and comprehensive laboratory panels do not contribute to risk stratification and should be reserved only for cases with clear clinical indicators or secondary trauma.

In older cohorts, this diagnostic framework is operationalized through repeated active standing protocols and systematic medication reconciliation. These steps capture transient, delayed, or initial blood pressure variations, postprandial drops, and iatrogenic volume depletion caused by polypharmacy. When the initial evaluation leaves the diagnosis uncertain, second-line investigations are deployed in a purpose-driven manner. Carotid sinus massage (CSM) is indicated for patients over 40 years of age, and therefore also in the elderly, with unexplained episodes compatible with a reflex mechanism, identifying cardioinhibitory carotid sinus syndrome when it reproduces spontaneous symptoms or documents prolonged ventricular pauses. Head-up tilt testing evaluates autonomic failure and exposes a general hypotensive susceptibility, guiding subsequent therapeutic modifications. Finally, the guidelines emphasize tailoring electrocardiographic monitoring to the frequency of symptoms, supporting the early implantation of loop recorders (ILRs) for patients with unexplained, recurrent episodes or unexplained falls without high-risk criteria. Implementing dedicated, multidisciplinary care pathways and outpatient syncope units shortens diagnostic delays and avoids inappropriate, costly hospitalizations while ensuring mechanism-based, individualized therapies for the older population.

All these indications of the guidelines remain valid and currently applicable in the elderly population in order to identify the correct pattern of syncope and rapidly direct the most appropriate therapy.

## 5. Risk Stratification in Geriatrics: ESC Guidelines High-Risk Features vs. Risk Scores

In older adults, the ESC 2018 Guidelines prioritize structured history, orthostatic blood pressure, and a 12-lead ECG to classify low- versus high-risk presentations and guide disposition within time-critical care pathways [3]. This feature-based approach performs well in geriatrics because prodromes, triggers, and ECG red flags remain interpretable even amid multimorbidity and reduces reliance on age alone.

Risk scores such as Canadian Syncope Risk Score (CSRS), Osservatorio Epidemiologico sulla Sincope nel Lazio (OESIL), and San Francisco Syncope Rule (SFSR) standardize documentation and may be useful, yet many inputs, such as age, anemia, and abnormal ECG, are prevalent in frail patients, inflating false positives and prompting avoidable admissions, eventually translating into suboptimal predictive accuracy and calibration [6,14]. Consistent with the ESC 2018 Guidelines, we also find that scores should not dictate admission decisions in isolation; they are best used as adjuncts in intermediate-risk cases and for audit across services.

Geriatric modifiers, including cognitive impairment, unwitnessed falls, anticoagulation, and recurrent trauma, influence short-term harm more than etiologic certainty and are not captured by most scores at presentation. Therefore, clinicians should pair the ESC 2018 Guidelines’ high-risk features with bedside assessments of frailty, mobility, and medication burden, then choose observation, targeted testing, or discharge with safety nets accordingly.

When a score is used, selecting one aligned with the clinical question helps: CSRS is appropriate for 30-day serious outcomes, whereas the FAINT score (based on history of heart Failure, history of cardiac Arrhythmia, Initial abnormal ECG, elevated pro B-type Natriuretic peptide, elevated high-sensitivity Troponin T) is most useful older individuals [4]. Ultimately, the ESC 2018 Guidelines framework remains relevant, but geriatric care benefits from explicit thresholds for injury risk, deprescribing, and follow-up monitoring, rather than universal score-driven triage alone. Whereas artificial intelligence and internet of things applications will translate into better and more timely decisions remains to be formally tested [15].

## 6. Geriatric Adaptations in Syncope Evaluation: Updating the 2018 ESC Guidelines Through Modern Diagnostic Testing

While the 2018 European Society of Cardiology (ESC) Guidelines historically established a baseline framework for syncope evaluation, their rigid diagnostic algorithms appear increasingly obsolete when confronted with contemporary, multi-parametric geriatric practice (Table 1). The foundational “core” evaluation championed in 2018—relying on standard, intermittent manual blood pressure cuffs during active standing—is fundamentally inadequate for the complex hemodynamic variations in the modern older patient. Recent evidence highlights that conventional sphygmomanometer frequently misses transient, immediate, or highly fluctuating blood pressure drops. Contemporary protocols now mandate the execution of continuous, beat-to-beat hemodynamic monitoring and standardized active standing pathways to capture initial, delayed, or postprandial hypotensive phenotypes that would otherwise remain completely undetected under the 2018 paradigm [10].

Furthermore, the 2018 guidelines’ reliance on static, in-office or in-hospital vital signs fails to address the dynamic nature of autonomic failure. Modern geriatric syndromic phenotyping has shifted toward the routine integration of 24-h Ambulatory Blood Pressure Monitoring (ABPM). This approach provides crucial, real-time data regarding nocturnal hypertension and daytime hypotensive burdens, which are vital for guiding safe iatrogenic deprescribing and aggressive medication reviews in frail individuals—a clinical granularity completely unaddressed by the retro 2018 recommendations [16].

The approach to second-line provocative testing has similarly outgrown the 2018 dictates. While the older guidelines vaguely outline carotid sinus massage (CSM), modern clinical practice demands a far more meticulous, safety-driven approach. The contemporary standard requires the adoption of the validated “Six-Step Method”—performed rigorously in both supine and upright positions under continuous, simultaneous ECG and non-invasive pressure monitoring—while strictly delineating absolute contraindications such as recent transient ischemic attacks, stroke, or suspected critical carotid stenosis [17].

Similarly, the role of head-up tilt testing has transitioned away from the simple, passive diagnostic binary endorsed in 2018. Today, tilt testing is utilized as a dynamic tool to document general hypotensive susceptibility, heavily augmented by continuous digital video recording. This technological addition optimizes the symptom-hemodynamic correlation, allowing clinicians to definitively differentiate true dysautonomic reflex syncope from psychogenic pseudosyncope, while rendering routine neuroimaging panels even more obsolete [18].

Perhaps the most glaring limitation of the 2018 ESC Guidelines lies in their slow, conservative tiering of cardiac rhythm monitoring. The retro strategy of prioritizing short-term telemetry or passive external Holter patches for infrequent events creates unnecessary diagnostic delays, leading to prolonged hospitalizations and recurrent physical trauma in the elderly. Contemporary digital electrophysiology supports an immediate shift toward the first-line deployment of remotely monitored Implantable Loop Recorders (ILRs) for recurrent, unexplained syncope in patients with low-to-intermediate short-term risk [19].

By integrating continuous remote monitoring pathways, modern clinical networks drastically shorten the time to actionable arrhythmia detection compared to the static follow-up schemes of the past. This proactive strategy directly streamlines the management of mobility-limited geriatric patients, ensuring that monitoring is selected precisely when it alters treatment, interfaces with fall-prevention frameworks, and coordinates directly with specialized, digitalized syncope units [5].

**Table 1:** Diagnostic tools for syncope, with pros and cons. Abbreviations and acronyms: ABPM: Ambulatory Blood Pressure Monitoring; ATP: Adenosine Triphosphate; BBB: Bundle Branch Block; BP: Blood Pressure; CSRS: Canadian Syncope Risk Score; CT: Computed Tomography; ECG: Electrocardiogram; ED: Emergency Department; EEG: Electroencephalogram; HBPM: Home Blood Pressure Monitoring; HTN: Hypertension; MI: Myocardial Infarction; MRI Magnetic Resonance Imaging; OESIL: Epidemiological Observatory On Syncope In Lazio; OH: Orthostatic Hypotension; PE: Pulmonary Embolism; PNES: Psychogenic Non-Epileptic Seizures; POTS: Postural Orthostatic Tachycardia Syndrome; PPS Psychogenic Pseudosyncope; SFSR: San Francisco Syncope Rule; TIA: Transient Ischemic Attack; VT: Ventricular Tachycardia.

Key References	ESC 2018 Recommendation	Clinical Cons/Risks	Clinical Pros (High Utility)	Diagnostic Tool
**FIRST-LINE/BEDSIDE EVALUATION**				
Jansen 2024 [1], de Ruiter 2018 [2], Brignole 2018 [3]	**Class I, Level C** *(Core starting point for all patients)*	• Limited by poor patient recall. • Cognitive impairment barriers. • Misses unwitnessed events.	• Highest diagnostic yield. • Rapid bedside phenotyping. • Anchors risk triage.	**Structured Initial Evaluation** *(History, Witness, Exam, ECG)*
Brignole 2018 [3], Dani 2021 [10]	**Class I, Level C** *(Indicated for suspected OH)*	• Misses short-lived drops. • High operator time burden. • Low compliance in frail cohorts.	• Immediate actionability. • Low cost and zero risk. • Identifies classic OH.	**Orthostatic BP/Active Standing** *(Manual Cuff)*
Brignole 2018 [3]	**Conditional** *(Only when clinically suspected)*	• High rate of false positives. • Overused indiscriminately. • Chronic disease confounding.	• Confirms specific triggers. • Identifies bleeding or hypoxia. • Refines cardiac risk.	**Targeted Laboratory Tests** *(Hb/Hct, Troponin, D-dimer)*
Brignole 2018 [3]	**Class I, Level C** *(For high-risk presentations)*	• Very low yield if unselected. • High resource utilization.	• Immediate arrhythmia safety net. • Captures early events.	**In-Hospital Telemetry**
**SECOND-LINE/SPECIALIZED TESTING**				
Brignole 2018 [3], de Lange 2024 [17]	**Class I, Level B** *(Diagnostically definitive if symptoms recur)*	• Common false positives. • Rare neurological injuries. • Avoid if prior TIA/stroke.	• Diagnoses carotid sinus syndrome. • Highly relevant over 40 years.	**Carotid Sinus Massage (CSM)**
Brignole 2018 [3], Cooper 2023 [18]	**Class IIa, Level B** *(For suspected reflex, OH, or POTS)*	• Limited rule-in value alone. • Resource and time intensive. • High rate of false negatives.	• Exposes hypotensive tendency. • Video-EEG links hemodynamics. • Rules out pseudosyncope.	**Head-Up Tilt Table Testing**
Brignole 2018 [3]	**Class I, Level B** *(When structural disease is suspected)*	• Low yield without baseline signs. • Syncope alone is not an indication.	• Confirms structural heart disease. • Rules out severe aortic stenosis. • Essential for risk scoring.	**Echocardiography (TTE)**
Brignole 2018 [3], Groppelli 2025 [16]	**Conditional** *(Class I B for autonomic failure;* *IIa C for OH/supine HTN monitoring;* *IIb C for suspected low BP during symptoms)*	• Patient compliance barriers. • Limited logistical availability.	• Tracks real-life diurnal shifts. • Reveals nocturnal hypertension. • Guides medication reviews.	**24 h ABPM/HBPM**
Brignole 2018 [3], Dani 2021 [10]	**Conditional (Class IIb, Level C)** *(Preferred if short drops suspected)*	• Scarce hospital availability. • Heavy motion artifacts.	• Captures transient initial OH. • Links symptom to pressure drop.	**Continuous Beat-to-Beat BP**
**CARDIAC RHYTHM MONITORING**				
Brignole 2018 [3]	**Class IIa, Level B** *(Only if frequent symptoms present)*	• Negligible yield if sporadic. • High cost per diagnosis.	• Widely available and low cost. • Useful if events are weekly.	**Holter Monitoring**
Brignole 2018 [3]	**Class IIa, Level B** *(If inter-symptom interval ≤4 weeks)*	• Requires active patient compliance. • Useless for rare events.	• Higher yield than Holter monitor. • Good if symptoms recur ≤4 weeks.	**External Loop Recorder (ELR)**
Brignole 2018 [3], Sulke 2016 [19], Russo 2023 [5]	**Class I, Level A** *(Early triage in recurrent unexplained syncope)*	• Requires minor surgical incision. • Risk of capturing incidentalomas.	• Highest cumulative diagnostic yield. • Guides mechanism-based therapy. • Highly cost-effective long-term.	**Implantable Loop Recorder (ILR)**
Brignole 2018 [3]	It is recommended without clear indication expressed.	• Requires prompt access and expertise; • may still need correlation with symptoms	• Rapidly identifies device malfunction/arrhythmic events; • may reduce inappropriate admissions	**Device interrogation (pacemaker/ICD)**
**MEDICAL IMAGING**				
Brignole 2018 [3], Burgese 2024 [20]	**Class III, Level B** *(Contraindicated/Avoid routinely)*	• Minimal yield in true syncope. • Unnecessary radiation and cost. • Risk of incidental findings.	• Crucial if focal neuro deficits. • Rules out structural lesions.	**Brain CT/MRI**
Brignole 2018 [3]	**Class III, Level B** *(Contraindicated/Avoid routinely)*	• Low yield for typical syncope. • Triggers cascade of cost/testing.	• Validated for stroke/TIA workup.	**Carotid Duplex Ultrasound**
Brignole 2018 [3]	**Class III, Level B** *(Contraindicated if syncope is likely)*	• Misleading false negatives. • Low value when syncope is likely.	• Identifies true epilepsy/seizures.	**Electroencephalogram (EEG)**
**EMERGING & FUTURE PARADIGMS**				
Probst 2020 [4], Voigt 2022 [6], Giamello 2026 [14]	**Class IIb, Level B** *(Optional in ED; never in isolation)*	• Over-triages older frail adults. • Does not beat clinical judgment.	• Standardizes ED documentation. • Useful for multi-center audits.	**Digital Risk Scores** *(FAINT, CSRS, OESIL)*
Charlton 2022 [21], Lee/Cho 2026 [22], González-Cañete 2021 [23]	**Not Included** *(Emerging frontier for geriatric update)*	• Strict data privacy concerns. • Technical literacy barriers. • Tech cost and compliance.	• Continuous real-time telemetry. • Pre-syncope AI alerts (84% yield). • Automated fall detection.	**Wearable Technologies** *(Smartwatches, PPG patches)*

## 7. Unmet Needs and Methodological Gaps in Geriatric Syncope Management

Despite containing a dedicated section on comorbidity and frailty, the 2018 European Society of Cardiology (ESC) Guidelines handle the geriatric population through a predominantly descriptive approach, lacking specific, action-oriented recommendations and standardized operational targets tailored to older individuals. A primary limitation lies in emergency department triage and risk-stratification pathways, which rely almost exclusively on cardiocentric metrics—such as ejection fraction and electrocardiographic anomalies—while completely omitting validated geriatric screening tools like the Clinical Frailty Scale (CFS) or assessments of functional independence (ADL: Activities of Daily Living/IADL: Instrumental Activities of Daily Living). This deficit extends into therapeutic optimization; although the guidelines generically advocate for medication reviews, they fail to provide structured deprescribing algorithms or integrate explicit geriatric criteria, such as the Beers or STOPP/START criteria (STOPP: Screening Tool of Older Persons’ Prescriptions and START: Screening Tool to Alert to Right Treatment) to systematically mitigate iatrogenic orthostatic hypotension and polypharmacy. Furthermore, the framework maintains a rigid diagnostic separation between transient loss of consciousness (TLOC) and accidental falls, ignoring the fact that age-related retrograde amnesia frequently misclassifies true syncopal episodes as unexplained falls, which underscores the urgent need for a unified clinical pathway. Additionally, current dynamic autonomic phenotyping remains heavily constrained by standard adult models that are insufficient for capturing fluctuating geriatric phenotypes, such as postprandial or delayed orthostatic hypotension, which require continuous beat-to-beat tracking or 24-h Ambulatory Blood Pressure Monitoring (ABPM) to guide safer treatment adjustments. Finally, current therapeutic recommendations are predominantly driven by device-based, instrumental algorithms that fail to address the complex ethical landscape of patients with advanced cognitive decline or dementia, leaving a critical gap in shared decision-making frameworks that balance overall life expectancy and advanced care planning against the singular goal of preventing syncope recurrences.

## 8. Evidence-Based Management of Geriatric Syncope: Wards to Devices

The 2018 ESC Guidelines endorse a mechanism-driven, stepwise approach that remains highly practical in geriatric medicine, provided it is operationalized through a standardized ‘syncope bundle’ upon admission. In geriatric wards, this bundle translates into an immediate initial assessment—comprising a witnessed history to overcome cognitive barriers, repeated orthostatic blood pressure measures, a 12-lead ECG, and targeted laboratory panels—coupled with systematic medication reconciliation performed by trained clinical staff [3,24]. Clinical disposition should be guided strictly by bedside high-risk features rather than chronological age alone, utilizing observation beds for intermediate cases, securing early cardiology input for electrocardiographic ‘red flags,’ and actively discouraging routine, low-yield neuroimaging or carotid duplex ultrasound [24]. Because polypharmacy frequently amplifies orthostatic drops and reflects underlying hypotensive susceptibility, down-titrating or deprescribing vasodepressors represents the primary therapeutic lever [3,10]. This iatrogenic optimization must be paired with structured, non-pharmacological interventions, including individualized hydration and salt optimization, compression garments, head-up sleeping, and supervised physical counterpressure maneuvers when the patient’s cognition and mobility safely allow [3,10].

When conservative lifestyle modifications fail and symptoms persist, pharmacological options like midodrine or fludrocortisone may be considered in carefully selected low-blood-pressure phenotypes; however, geriatric practice dictates conservative dosing titration; strict surveillance for supine hypertension, urinary retention, or electrolyte disturbances; and regular reassessments to avoid heart failure exacerbations [10,25]. If the initial evaluation remains non-diagnostic, diagnostic escalation should follow a protocolized pathway: inpatient telemetry is prioritized for high-risk presentations, Holter or external loop recorders are reserved for frequent episodes, and early implantable loop recorder (ILR) integration is preferred for recurrent, unexplained syncope or unexplained falls [26,27]. For reflex syncope with documented asystolic pauses, current evidence—including post-2018 trials of dual-chamber pacing with closed-loop stimulation (DDD-CLS)—strongly supports permanent pacing in chosen older adults, though clinicians must recognize that a dominant vasodepressor component can blunt device benefit, warranting parallel blood-pressure strategies [3,27,28]. Conversely, in suspected arrhythmic syncope, management directly follows the underlying substrate. An expert arrhythmology evaluation is highly warranted in geriatric patients presenting with borderline conduction disease, such as bifascicular block, where an empirical pacemaker implantation is often preferred to achieve rapid symptom control and minimize trauma risk, whereas younger cohorts typically undergo expectant monitoring or electrophysiological studies [21,27]. Ultimately, future advanced interventions like cardioneuroablation remain strictly investigational for the majority of geriatric patients [29]. Successful implementation of this framework within the care pathway relies on a multidisciplinary approach, auditable quality metrics, comprehensive caregiver education regarding trauma precautions, and the integration of remote device monitoring to substantially shorten the time to a definitive diagnosis [5,26] (Table 2).

## 9. What Has Changed Since 2018? Evidence Updates That May Shift Practice

Since the publication of the 2018 ESC Guidelines, the clinical evidence base has undergone key paradigm shifts, particularly regarding device-based interventions and pharmacological tailoring in the elderly. A major evolution from the 2018 framework is the robust expansion of evidence favoring pacemaker therapy for selected reflex syncope presentations with tilt-induced asystole; specifically, contemporary data have established that dual-chamber pacing with closed-loop stimulation (DDD-CLS) significantly lowers recurrences in adults over 40, directly shifting how clinicians guide device-implantation discussions in frail older adults [1,31]. Furthermore, regarding older patients presenting with bifascicular block and unexplained syncope, head-to-head comparisons of empiric pacing versus implantable monitoring have refined the 2018 assumptions; while empiric pacing offers faster symptom control, recent insights warn of the substantial risk of treating non-arrhythmic mechanisms, thereby mandating a much more rigorous pre-implantation phenotyping than previously recommended [27].

Parallel to this, the diagnostic timeline has evolved beyond the conservative, tiered approach of 2018 toward the early deployment of Implantable Loop Recorders (ILRs). Recent clinical series demonstrate a high cumulative diagnostic yield with early ILR use, enabling definitive symptom–rhythm correlation before committing to permanent therapy—a strategy that represents a vital advancement when age-related cognitive impairment limits the accuracy of the clinical history [32]. This diagnostic acceleration is further transformed by the integration of remote ILR monitoring, which has drastically shortened the time to actionable arrhythmia review compared to the static follow-up schemes of 2018, thereby streamlining specialized pathways that reduce hospital visits and accelerate critical decisions regarding anticoagulation, pacing, and iatrogenic medication adjustments [5].

Pharmacological management has evolved significantly beyond the general recommendations of the 2018 text. Recent meta-analyses have solidified the role of midodrine in reducing vasovagal recurrences, while the contemporary routine use of 24-h ambulatory blood pressure monitoring (ABPM) has redefined clinical practice by identifying specific low-pressure phenotypes that are highly likely to benefit from active pressor therapy rather than standard deprescribing alone [33]. For orthostatic and postprandial hypotension, modern evidence has superseded the generic lifestyle advice of 2018 by supporting individualized fluid and salt targets, advanced compression strategies, and cautious pressor titration seamlessly integrated with multifactorial fall-prevention frameworks, effectively extending the foundational principles of the 2018 ESC Guidelines into precise, ward-level geriatric practice [34].

The clinical efficacy of cardioneuroablation (CNA) has been progressively documented across several clinical cohorts, showing promising results in selected populations. Evidence from published observational studies indicates that CNA achieves a highly satisfactory syncope-free rate ranging from 73.2% to 100% [35,36,37]. Despite the historical reliance on non-randomized data, more robust evidence has recently emerged from the first prospective randomized controlled trial on this topic [30]. In this study of 48 patients, CNA demonstrated a stark superiority over optimal non-pharmacological conservative management, yielding a 92% efficacy rate compared to just 46% in the control arm (*p* = 0.0004). However, translating these high success rates into the geriatric population poses significant clinical and pathophysiological challenges. The vast majority of published clinical trials and observational data exclusively represent younger cohorts under the age of 60 [30,37]. In older individuals (age > 60 years), procedural efficacy appears to be notably attenuated. For instance, clinical investigations have reported a significantly lower post-ablation heart rate increase and a lack of meaningful improvements in overall quality of life among older patients [38,39]. According to the recent international consensus statement from major heart rhythm societies [29], the restricted utility of CNA in older adults is strongly driven by age-related cardiovascular variations. A primary concern in this demographic is the frequent coexistence of a “dual mechanism”. While younger patients typically present with functional, pure extrinsic vagal responses, older individuals almost invariably exhibit a subtle degenerative process of the intrinsic sinoatrial node or the atrioventricular conduction system that overlaps with vagal hyperactivity [29]. Because CNA solely targets the parasympathetic autonomic plexi, its therapeutic power is fundamentally compromised when underlying structural conduction disease is present. Furthermore, older patients suffering from reflex syncope frequently present with a dominant vasodepressor or low-blood-pressure phenotype rather than a cardioinhibitory one [40]. The expert writing committee explicitly underscores that parasympathetic cardiac denervation is completely inappropriate for addressing these hypotensive components, as it exerts no physiological control over peripheral vascular failure or acute baroreflex dysfunction [29]. Given the absolute lack of dedicated randomized controlled trials in older cohorts, the clinical status of CNA for patients aged > 60 years remains strictly investigational. Consequently, current expert recommendations advocate that conventional cardiac pacing should remain the standard-of-care for elderly patients with severe bradyarrhythmic manifestations, whereas CNA must be restricted to controlled clinical trial settings for this specific demographic [24,29].

Moreover, in the clinical evaluation of older adults, the integration of non-invasive wearable devices, such as smartwatches and patch monitors, represents a rapidly emerging diagnostic frontier. Despite these devices being mainly tested for atrial fibrillation detection, they could represent a valid diagnostic tool for syncope. Syncope in elderly cohorts is frequently characterized by sudden episodes lacking predictable prodromal symptoms, carrying a highly critical risk of physical trauma, fractures, and hospital admissions. Traditional ambulatory electrocardiogram monitoring systems, such as 24-to-48-h Holter monitors, are fundamentally limited by narrow recording windows that often fail to capture intermittent or paroxysmal events. Conversely, consumer wearables facilitate long-term, continuous, and non-invasive tracking of crucial cardiovascular metrics outside of classical hospital environments [41]. By leveraging built-in photoplethysmography (PPG) sensors and inertial measurement units, these smart devices monitor real-time heart rate, heart rate variability (HRV), and sharp postural shifts [41]. Robust data from prospective observational clinical trials have recently demonstrated that artificial intelligence and machine learning models applied to smartwatch PPG metrics can accurately identify impending neurocardiovascular alterations. Specifically, these algorithms are capable of predicting vasovagal syncope episodes up to five minutes before a transient loss of consciousness occurs, achieving an overall accuracy rate of 84.6% during head-up tilt testing [22]. Furthermore, large-scale pragmatic trials and consumer-led validation cohorts have established a precise symptom-rhythm correlation that is essential for diagnosing underlying channel conduct defects, vasospastic triggers, or silent arrhythmogenic conditions like atrial fibrillation [41]. Newly engineered continuous wearable blood pressure sensors also provide an actionable framework to detect inappropriate short-term blood pressure variability and acute hypotensive drops, which are key physiological markers of age-related baroreflex dysfunction and orthostatic disorders. For an elderly patient, such real-time biometric tracking acts as an early warning system, allowing them to proactively adopt safe, recumbent positions before a syncopal event takes place [22]. In cases where profound hemodynamic compromise results in an unpreventable fall, real-world data from digital health registries confirm that integrated automated detection algorithms utilizing tri-axial accelerometers and gyroscopes can immediately calculate the impact force, recognize the posture state, and transmit immediate distress signals alongside telemetry data to caregivers or emergency health services [23,42,43,44]. Despite these diagnostic advantages, notable barriers to adoption persist among elderly users, driven by limited digital literacy, cognitive or physical impairments, and cost-related concerns [41,42]. Consequently, maximizing the clinical utility of wearables in geriatric care requires age-friendly design and individualized support to improve adherence and ensure home-based safety.

## 10. Knowledge Gaps and Future Directions

Despite the foundational framework established by the 2018 ESC Guidelines, geriatric syncope remains critically under-studied because multi-morbidity, frailty, and cognitive impairment have historically served as systematic exclusion criteria in major clinical trials, severely limiting the external validity of traditional diagnostic algorithms in routine practice [20,45]. Key contemporary knowledge gaps include the lack of standardized operational protocols for assessing fluctuating orthostatic and postprandial hypotension, undefined optimal blood pressure thresholds during iatrogenic deprescribing, and a poor understanding of how aggressive arrhythmia monitoring interfaces with competing geriatric risks, such as devastating falls and bleeding complications. To overcome these limitations, future research must move beyond the retro, feature-based triage of 2018 and validate contemporary risk scores that embed pragmatic, patient-centered endpoints—such as functional decline, recurrent traumatic injury, and institutionalization rates—while formalizing shared decision-making and caregiver perspectives across diverse care settings. Furthermore, precision mechanistic phenotyping must be strengthened by integrating 24-h ambulatory blood pressure monitoring (ABPM) and continuous, beat-to-beat hemodynamic tracking to replace static manual cuff measures, coupled with cost-effectiveness analyses tailored specifically to frail inpatients and community-dwelling older cohorts.

Crucially, the future of geriatric syncope management lies in the large-scale integration of consumer- and medical-grade wearable devices coupled with advanced telemedicine platforms. Future clinical frameworks must validate the use of smartwatch-derived photoplethysmography (PPG) and artificial intelligence algorithms to establish real-time, home-based early warning systems capable of predicting transient loss of consciousness before hemodynamic collapse occurs. Additionally, telemedicine infrastructure must be expanded to seamlessly transmit continuous sensor data and automated fall-detection metrics directly to specialized syncope units. This digital network will be vital to drastically shorten the time to actionable diagnosis, streamline remote medication reviews, and provide a continuous safety net for mobility-limited older adults in their home environments. Finally, large-scale, randomized, age-stratified trials remain urgently required to refine the exact indications for dual-chamber pacing with closed-loop stimulation (DDD-CLS) and emerging invasive interventions like cardioneuroablation while establishing precise, multi-parametric protocols for combining safe deprescribing with targeted pressor therapies (such as midodrine or fludrocortisone) to eliminate supine hypertension while preserving autonomy and overall quality of life.

## 11. Conclusions

**Figure 1:**
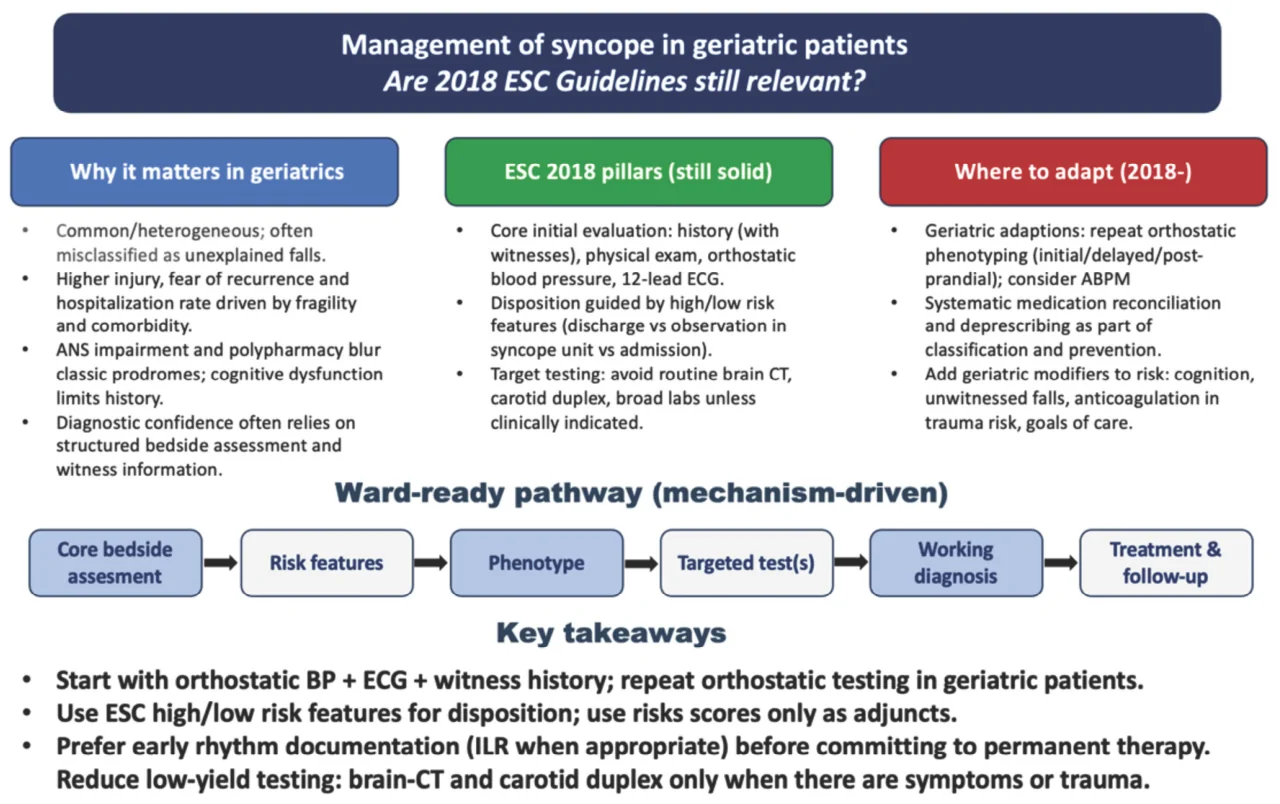
Modern management of syncope in the elderly. ABPM = ambulatory blood pressure monitoring; ANS = autonomic nervous system; BP = blood pressure; CT = computed tomography; ESC = European Society of Cardiology; ILR = implantable loop recorder.

This review provides a comprehensive, critical synthesis of the evolving landscape in geriatric syncope management, balancing established clinical protocols with emerging digital paradigms up to 2026 (Figure 1). The primary strength of this work lies in its specific and dedicated focus on the older patient cohort, mapping the intricate intersection between multi-system cardiovascular aging, polypharmacy, and frailty—variables that have historically been neglected or systematically excluded from major clinical trials. Furthermore, by successfully contrasting traditional diagnostic frameworks with novel, non-invasive digital solutions and automated fall-detection metrics, this review offers a pragmatic, forward-looking perspective on home-based safety networks.

When addressing the central clinical query of this investigation—whether the 2018 ESC Guidelines remain applicable in the modern era—the answer requires a nuanced, dual perspective. The foundational principles of the 2018 framework, specifically the emphasis on a structured initial evaluation (careful history with witness accounts, orthostatic vital signs, and standard ECG) and the mandate for a mechanism-driven therapeutic triage, remain undeniably valid and clinically durable. They continue to serve as the vital initial safeguard against high-risk cardiac events in geriatric wards. However, when evaluated against the clinical and technological standards of 2026, the specific operational methods of the 2018 text appear increasingly obsolete and retro. Static manual blood pressure cuffs, slow and conservative monitoring tiers, and the omission of validated frailty or deprescribing metrics are fundamentally inadequate for the hemodynamic complexity of the modern older adult. The 2018 guidelines provide a rigid baseline, but they fail to address the dynamic autonomic fluctuations and the need for immediate, actionable diagnostics required in contemporary practice. Analytically, the management of geriatric syncope can no longer be confined to a purely cardiocentric or hospital-centric model. The traditional boundaries between transient loss of consciousness and unexplained falls must be permanently dismantled through unified, interdisciplinary clinical pathways. The clinical trajectory is rapidly shifting from reactive, device-based interventions toward proactive, continuous, and non-invasive biometric phenotyping. While definitive pharmacological protocols and large-scale, age-stratified randomized controlled trials are still urgently required to validate emerging interventions like cardioneuroablation in older cohorts, the immediate future belongs to the synergy between telemedicine and artificial intelligence-driven wearable devices. Integrating smart sensors into everyday geriatric care will not only bridge the diagnostic gaps left by the 2018 guidelines but will also ultimately achieve the dual clinical goal of maximizing diagnostic efficiency and preventing secondary physical trauma, thereby preserving the functional autonomy and overall quality of life of the aging population.

## Figures and Tables

**Table 2:** Therapeutic Interventions in Geriatric Syncope: Mechanism, ESC 2018 Class, and Modern Clinical Practice. Abbreviations and acronyms: DDD-CLS dual-chamber, rate-adaptive cardiac pacing mode that uses Closed Loop Stimulation; HF heart failure; OH: Orthostatic Hypotension.

Key References	ESC 2018 Recommendation	Clinical Cons/Geriatric Risks	Clinical Pros (High Utility)	Therapeutic Strategy
**VOLUMETRIC & BEHAVIORAL INTERVENTIONS**				
Brignole 2018 [3], Dani 2021 [10], Groppelli 2025 [16]	**Class I, Level C** *(First-line for drug-induced OH/reflex)*	• Risk of rebound hypertension. • Can worsen heart failure. • Requires close primary care links.	• Targets primary iatrogenic cause. • Low cost and zero device risk. • Reduces polypharmacy burden.	**Iatrogenic Deprescribing** *(Down-titration of vasodepressors)*
Brignole 2018 [3], Groppelli 2025 [16]	**Class I, Level C** *(Indicated if no fluid restrictions)*	• Contraindicated in severe HF. • Exacerbates chronic kidney disease. • Poor compliance in cognitive decline.	• Non-invasive volume expansion. • Zero pharmacological cost. • Empowers patient self-care.	**Hydration & Salt Optimization** *(Fluid load, dietary adjustments)*
Brignole 2018 [3]	**Class IIa, Level B** *(If prodromal symptoms are present)*	• Useless if prodromes are absent. • Restricted by severe frailty. • High risk of locomotor falls.	• Aborts impending reflex syncope. • Immediate active pressure rise. • Zero cost.	**Physical Counterpressure Maneuvers** *(Leg crossing, hand grip)*
Brignole 2018 [3], Groppelli 2025 [16]	**Class IIa, Level C** *(Highly recommended for OH)*	• Very difficult to put on alone. • High skin breakdown risk. • Poor long-term patient compliance.	• Prevents venous pooling. • Effective for postprandial drops. • Combats gravitational stress.	**Mechanical Compression** *(Abdominal binders, stockings)*
**PHARMACOLOGICAL TREATMENT**				
Brignole 2018 [3], Dani 2021 [10]	**Class IIa, Level B** *(For refractory OH or reflex phenotypes)*	• High risk of supine hypertension. • Triggers urinary retention. • Requires conservative low dosing.	• Increases peripheral resistance. • Proven reduction in recurrences. • Rapid onset of action.	**Midodrine** *(Alpha-1 adrenergic agonist)*
Brignole 2018 [3], Dani 2021 [10]	**Class IIa, Level C** *(Mainly for OH without heart failure)*	• Induces severe hypokalemia. • Promotes fluid overload/edema. • High risk of worsening HF.	• Potent plasma volume expansion. • Sustained pressure support.	**Fludrocortisone** *(Mineralocorticoid)*
**DEVICE-BASED & INVASIVE THERAPIES**				
Brignole 2018 [3], Piotrowski 2023 [30]	**Class IIa, Level B** *(For documented cardioinhibitory reflex)*	• Invasive surgical procedure. • Risk of device lead infection. • Ineffective if vasodepression rules.	• Dynamic response to vagal surges. • Directly limits asystolic drops. • Exceptional results over 40 years.	**DDD-CLS Pacing** *(Closed-Loop Dual Chamber)*
Brignole 2018 [3], Sulke 2016 [19]	**Class IIa, Level B** *(Optional if no mechanism found)*	• High rate of unnecessary implants. • Misses non-arrhythmic mechanisms. • Induces pacing-induced RV pacing.	• Immediate bradycardia protection. • Lowers sudden syncopal trauma. • Avoids multiple ED admissions.	**Empiric Pacing** *(In Bifascicular Block)*
Francia 2023 [25], Aksu 2024 [29]	**Not Included** *(Considered strictly investigational)*	• Strictly experimental over 60. • Blunted heart rate rise in elders. • Fails against intrinsic node disease.	• Permanent cure for vagal block. • Eliminates need for pacemakers. • 91.9% overall success rate.	**Cardioneuroablation (CNA)** *(Autonomic denervation)*
Brignole 2018 [3]	**Class I, Level A** *(Standard indications for cardiac syncope)*	• High risk of inappropriate shocks. • Aggravates end-of-life distress. • High peri-procedural complication rate.	• Prevents arrhythmic sudden death. • Mandatory for ischemic substrate.	**ICD Implantation** *(Cardioverter-Defibrillator)*

## Data Availability

No new data were created or analyzed in this study. Data sharing is not applicable.
